# Staff- and service-level factors associated with organisational readiness to implement a clinical pathway for the identification, assessment, and management of anxiety and depression in adults with cancer

**DOI:** 10.1186/s12913-023-09829-2

**Published:** 2023-08-15

**Authors:** Mona M. Faris, Heather L. Shepherd, Phyllis N. Butow, Patrick Kelly, Sharon He, Nicole Rankin, Lindy Masya, The ADAPT Program Group, Joanne Shaw

**Affiliations:** 1 Psycho-Oncology Co-Operative Research Group (PoCoG), School of Psychology, The University of Sydney, Sydney, NSW 2006 Australia; 2 Susan Wakil School of Nursing and Midwifery, Faculty of Medicine and Health, The University of Sydney, Sydney, NSW 2006 Australia; 3 School of Public Health, The University of Sydney, Sydney, NSW 2006 Australia; 4 Faculty of Medicine and Health, The University of Sydney, Sydney, NSW 2006 Australia

**Keywords:** Organisational readiness, Implementation science, Organizational Readiness for Implementing Change scale, Psycho-oncology, Cluster randomised controlled trial

## Abstract

**Background:**

Organisational readiness is recognised as a key factor impacting the successful translation of research findings into practice. Within psycho-oncology, measuring organisational readiness and understanding factors impacting organisational readiness is crucial as it is often challenging to implement evidence-based findings into routine cancer care. In this quantitative study, we examined the level of organisational readiness of cancer services preparing to implement a clinical pathway for the screening, assessment, and management of anxiety and depression in adult cancer patients (the ADAPT CP) within a cluster randomised controlled trial and sought to identify staff- and service-level factors associated with organisational readiness.

**Methods:**

Multidisciplinary staff across 12 Australian cancer services were identified. Their perceptions of their services’ readiness to implement the ADAPT CP in the cancer stream or treatment modality selected within their service was assessed prior to implementation using the Organizational Readiness for Implementing Change scale. Data collection included staff demographic and professional characteristics, and their perception of the ADAPT CP using a set of 13 study-specific survey items. Service characteristics were captured using a site profile audit form and workflows during site engagement.

**Results:**

Fourteen staff- and service-level factors were identified as potentially impacting organisational readiness. To identify factors that best explained organisational readiness, separate univariate analyses were conducted for each factor, followed by a backward elimination regression. Compared to services that implemented the ADAPT CP in one treatment modality, those opting for four treatment modalities had significantly higher organisational readiness scores. Staff in administrative/technical support/non-clinical roles had significantly higher organisational readiness scores compared to psychosocial staff. Higher organisational readiness scores were also significantly related to more positive perceptions of the ADAPT CP.

**Conclusions:**

Readiness to implement an anxiety and depression clinical pathway within 12 oncology services was high. This may be attributed to the extensive engagement with services prior to implementation. The factors associated with organisational readiness highlight the importance of ensuring adequate resourcing and supporting staff to implement change, effectively communicating the value of the change, and taking a whole-of-service approach to implementing the change. Future longitudinal studies may identify factors associated with ongoing readiness and engagement prior to implementation.

**Trial registration:**

The ADAPT RCT was registered prospectively with the ANZCTR on 22/03/2017. Trial ID ACTRN12617000411347. https://www.anzctr.org.au/Trial/Registration/TrialReview.aspx?id=372486&isReview=true.

**Supplementary Information:**

The online version contains supplementary material available at 10.1186/s12913-023-09829-2.

## Introduction

Many effective evidence-based innovations (e.g., interventions, guidelines, clinical pathways) have been developed, yet, translating evidence into practice is often slow; it takes on average 17 years for evidence-based interventions to become part of routine health care [1]. More than 60 models, theories, and frameworks have been developed to guide translation of evidence into practice [2], and to predict or explain how potential facilitators and barriers may impact the translation of research findings into practice [3].

One such facilitator, recognised as a key factor impacting implementation in a number of frameworks, is organisational readiness for change [4, 5]. Organisational readiness has been defined and measured in different ways [6]. Some definitions focus on the psychological and behavioural preparedness of individual members within an organisation [5]. Others focus on the collective attitude or perception that the organisation is willing and able to implement the change [7, 8]. Here, we employ the latter definition because implementation of a clinical pathway within health services is a collective effort, as clinical pathways require support, facilitation, and endorsement from senior managers or leaders, support from technical staff, the coordination of roles, and collective decision-making [4]. Furthermore, successful implementation is likely to lead to collective benefits such as improvements in the organisation’s workflows, productivity, and evidence-based care [4].

When organisational readiness is high, individuals within the organisation are more motivated to implement the change, and display greater persistence when faced with challenges [9]. Holt et al. [8] proposed that organisational readiness is influenced by the content of the change, the process by which the change is being implemented, the context in which the change is occurring, and the characteristics of individuals being asked to implement the change. Measuring organisational readiness and understanding factors that can impact organisational readiness are crucial as this allows change advocates to identify ways to prepare for change [8, 10]. This is particularly important within psycho-oncology given the documented challenges of implementing evidence-based psychosocial interventions in routine cancer care [11]. Yet, studies investigating factors that impact organisational readiness in psycho-oncology are lacking.

To address this gap, our team recently examined factors associated with organisational readiness for implementing a clinical pathway for the screening, assessment, and management of anxiety and depression in adult cancer patients (the ADAPT CP) [12]. Nominated staff at each service were responsible for introducing patients to screening, registering patients on the ADAPT Portal [13], following up with patients to discuss screening results and provide appropriate support where required, and monitor the uptake of referrals. The aims, methods, and measures used in the ADAPT CP trial have been described elsewhere [14, 15]. Using a mixed-methods approach, prior to the implementation of the ADAPT CP, organisational readiness was measured using the Organizational Readiness for Implementing Change (ORIC) survey [16]. The study provided an interim analysis based on data from 6 out of the 12 cancer services where the ADAPT CP was implemented. Services were categorised as having mid- or high-range organisational readiness based on ORIC scores, and semi-structured interviews explored differences in staff perceptions of service culture and characteristics between these two groups [12]. Staff at services reporting higher organisational readiness described the work culture as more collaborative, with clear communication, greater role flexibility, and adaptability to change behaviour.

The current study extends on the interim analysis by: 1) examining organisational readiness across all 12 services, and 2) using a quantitative approach to assess a range of factors that may impact organisational readiness. The ORIC allowed us to examine two of the four factors proposed by Holt et al. [8] as being influential for organisational readiness, namely, the context in which the change is implemented and characteristics of individuals being asked to implement the change. The primary research questions for the study were:

1. What is the level of organisational readiness of the 12 cancer services prior to the implementation of the ADAPT CP, as measured by the ORIC?
2. Which staff- and service-level factors are associated with perceived readiness to implement the ADAPT CP?

Previous findings suggest that an adequate number of psychosocial staff, having procedures in place for the provision of psychosocial care (e.g., processes for identifying the psychosocial needs of patients), and being trained in providing psychosocial care are perceived as facilitators to implementing psychosocial programs within oncology services [17, 18]. As the ADAPT CP focused on assisting services in identifying and managing anxiety and depression, we hypothesised that factors relating to the psychosocial care of patients (e.g., screening for psychosocial issues and having an adequate number of trained psychosocial staff) were likely to emerge as significant predictors of organisational readiness.

## Method

### Study design and setting

The ADAPT cluster randomised controlled trial (RCT) was implemented in 12 cancer services across New South Wales (NSW), Australia over a 12-month period. Sites were recruited between November 2017 and December 2020. The primary aim of the cluster RCT was to evaluate two implementation strategies (core versus enhanced) to determine the level of implementation effort required to successfully introduce the ADAPT CP. Participating services were cluster randomised, stratified by service size, to the core versus enhanced implementation strategy which was utilised by the research team for 12 months during which services implemented the ADAPT CP as part of routine care. Data for the primary study are reported elsewhere [19]. Refer to the Data analysis section for the full list of staff- and service-level predictors examined in this study.

### Participating services and staff

Eligible services were those that provide cancer care to at least 100 patients per year in New South Wales (NSW), Australia, and operate in a public and/or private healthcare system. Participating sites could be whole services or selected departments within those services (e.g., tumour streams or treatment modalities). Services were excluded if they were unable to commit to the study process (e.g., endorsing and enabling staff to participate in training for the ADAPT CP, engaging in a 3-month tailoring period prior to implementation), or did not have Wi-Fi/broadband/internet to support the use of a web-based portal that had been created to support implementation (ADAPT Portal).

Study participants were staff at the 12 cancer services who were employed on an ongoing (≥ 6 months) basis. Staff in roles that provide clinical, administrative, or technical/managerial support to the cancer service were identified through an organisational chart mapping exercise during site engagement and were invited to participate in this study and give informed consent. Participants included staff who would or would not be directly involved in the application of the ADAPT CP, to gauge a range of perceptions from a broader organisational perspective.

### Study procedure

In each service prior to implementation, one or more champion(s) was identified, and a multidisciplinary lead team was appointed, who engaged with the research team during 6–8 engagement meetings to tailor the ADAPT CP according to service resources, preferences, and existing workflows. Services also decided which treatment modality (e.g., medical oncology department, surgical oncology department) or streams (e.g., breast cancer, haematology) to implement the ADAPT CP. Staff were then provided with training to familiarise them with the ADAPT CP and the ADAPT Portal.

An email containing a link to a REDCap [20, 21] survey exploring perceptions of and attitudes to implementing the ADAPT CP was sent to participating staff members following the engagement period just before implementation of the ADAPT CP commenced (T0), again at 6 months (T1) and at 12 months (T2). The data analysed in this study is from the T0 staff survey responses only. To optimise response rates, two email reminders with the survey link were sent one week apart to cancer service staff [20, 21].

### Measures

#### Staff readiness, attitudes and characteristics

The Organizational Readiness for Implementing Change (ORIC) scale [16] was used to assess readiness to implement the ADAPT CP. The 12 item-scale assesses participants’ perception of their organisation’s commitment, motivation, and determination to change as well as the perceived level of support available and confidence in implementing the change. The original ORIC items were modified to focus respondents’ attention to implementation of the ADAPT CP based on the recommendations of the instrument authors [4]. For example, the item “People who work here are motivated to implement this change” was modified to “People who work here are motivated to implement the anxiety and depression pathway”. Respondents indicated their level of agreement with each statement using a 5-point Likert scale ranging from disagree to agree. Total scores could range from 12 to 60, with higher scores indicating greater perceived organisational readiness.

Perceptions of the ADAPT CP were captured using a set of 13 study-specific items assessing the: perceived need for and importance of an anxiety and depression clinical pathway (CP) for patients and for the organisation, credibility of the evidence-based ADAPT CP and the research team, perceived workload/burden of implementing the ADAPT CP, as well as perceived support from leaders and the availability of resources required to implement the ADAPT CP. Level of agreement with each statement was indicated using a 5-point Likert scale ranging from strongly agree to strongly disagree. Based on a factor analysis conducted on these items [22], two stable factors were identified, namely, *perceived benefit* (score out of 35) and *perceived burden* (score out 20) of the CP. Survey respondents’ scores for these factors were calculated.

Individual demographic and professional characteristics were also elicited. Information collected included age, gender, role, employment status, and years in current employment.

#### Site characteristics and workflows

Champions at each service completed a site profile audit to capture information about characteristics of the service. Information collected of relevance to the current study included site location (inner regional, major city), service type (public, private or mixed), hospital size as determined by the number of new patients over a 12-month period (< 100, ≥ 100), history of psychosocial screening (yes, no), and clinical load of psychosocial staff (number of new patients per 1.0 full-time equivalent of psychosocial staff). Decisions pertaining to the number of streams (1, 2, ≥ 3) and treatment modalities (1–4; medical, radiation, surgical, or haematological oncology) in which to implement the ADAPT trial were captured in the workflows during the site engagement meetings with team leaders (for more detail, see: [23]).

The study was approved by the Sydney Local Health District Human Research Ethics Committee, Protocol X16-0378 HREC/16/RPAH/522.

### Data analysis

Descriptive statistics in the form of means, frequencies, and proportions were used to summarise service and staff characteristics, and the ORIC and additional study-specific survey item responses.

To determine how the ORIC items would be grouped and used in the analyses, the latent structure of the ORIC was assessed in an exploratory factor analysis. Factors were extracted if they had an eigenvalue > 1 and item loadings were identified using a varimax rotation (with Kaiser normalisation). For ease of interpretability of the rotated factor loadings, an item was considered to load onto a factor if it loaded at least 0.40, and was less than 0.40 for the other factors [24].

Based on the literature and an understanding of the services implementing the ADAPT CP, a number of staff- and service-level factors were identified as having potential to influence staff perceptions of their service’s readiness to implement the ADAPT CP. The staff-level factors identified were: age, gender, role, employment status, length of time in current role, and each survey respondents’ *perceived benefit* and *perceived burden* score. The service-level factors identified were: site location, funding type, size, number of streams and treatment modalities that the ADAPT CP was implemented in, history of psychosocial screening, and clinical load of psychosocial staff.

For regression analyses, a minimum of 10 participants per predictor is required to produce reliable results [25]. In the current study, there are 14 predictors but a sample size < 140. To identify the predictors best able to explain the outcome variable and in turn, reduce the number of predictors, separate univariate regression analyses were run with the total ORIC score as the outcome variable and each of the staff- and service-level factors as a predictor variable. Predictors were retained if they had a significant relationship with the outcome variable (*p* < 0.25).

With the aim of developing a model with predictors that provide the best explanation for the outcome variable, a backward elimination regression was conducted. The initial, full model contained all significant predictors from the univariate regression analyses with variables eliminated from the model, starting with the predictor having the highest non-significant p-value (i.e., *p* > 0.05). The analysis adjusted for differences between services.

The assumptions of multiple regression were also checked. The REDCap survey data was extracted using Microsoft Excel. All analyses were performed using Stata, version 17.0.

## Results

### Site characteristics

Of the 12 cancer services, nine were located within a major city and three in inner regional areas (Table 1). Ten services were publicly funded, one was privately funded, and another was both a publicly and privately funded service. Services were stratified by size, with four services classified as small (< 100 new pts./year) and eight classified as large (≥ 100 new pts./year). Five services implemented the ADAPT CP in a single treatment modality, with other services implementing the ADAPT CP across two (*n* = 2) or more (*n* = 5) treatment modalities. Five cancer services indicated they had existing psychosocial screening processes in place prior to implementing the ADAPT CP. Clinical load of psychosocial staff varied across the services.

**Table 1:** Site characteristics

Site ID	Site location	Service funding type	Number of new patients seen per year	Treatment modalities included	Number of treatment modalities included	Tumour streams included	Number of streams included	Full-time equivalent (FTE) of Psychosocial staff	Screening history in past 12 months	Clinical load^a^
1	Major city	Public	≥ 100	Medical Radiation Haematology	3	All	≥ 3	0.8	Yes	14 085
2	Inner regional	Public	< 100	Medical Radiation Haematology Surgical	4	All	≥ 3	0.6	No	6 020
3	Inner regional	Public	< 100	Medical	1	All	≥ 3	0.6	No	11 280
4	Major city	Public	≥ 100	Medical Surgical	2	Gastro-intestinal	1	2.4	No	8 580
5	Inner regional	Public	< 100	Medical Radiation Haematology	3	All	≥ 3	1	Yes	4 452
6	Major city	Public	≥ 100	Medical Haematology	2	All	≥ 3	7.9	No	1 401
7	Major city	Public	≥ 100	Surgical	1	Upper gastro-intestinal	1	2.4	Yes	685
8	Major city	Public	< 100	Medical Radiation Haematology	3	All	≥ 3	5	Yes	3 797
9	Major city	Public	≥ 100	Haematology	1	Lymphoma, acute leukemia, multiple myeloma	≥ 3	2.4	No	685
10	Major city	Public	≥ 100	Medical Radiation Surgical	3	Head & Neck	1	4	No	4 596
11	Major city	Public and Private	≥ 100	Medical	1	Sarcoma, Gynae	2	6.9	Yes	4 480
12	Major city	Private	≥ 100	Medical	1	All	≥ 3	0.9	No	7 333

^a^This column represents the number of new patients per 1.0 FTE of psychosocial staff. This was calculated by dividing the number of new patients registered at a cancer service within a financial year [26] by the FTE of psychosocial staff at the cancer service

### Survey responses

There were 139 staff surveys returned at T0, with response rates across sites ranging from 13–40% (median = 27%). These response rates reflect high staff turnover, staff being on leave, and clinical workload. Of the returned surveys, 33 were excluded due to: missing ORIC items (*n* = 17), late responses (T0 survey completed after T1 or T2 survey was distributed) which are unlikely to reflect perceptions and attitudes held at T0 (*n* = 3), or a postcode entered not matching any participating services (*n* = 13), making it impossible to assess effects of site-level predictors on organisational readiness. Therefore, data analysis was based on analysis of the 106 completed surveys.

### Staff characteristics

Staff demographic and professional information is presented in Table 2. Survey respondents were predominantly female (86%) and aged between 26–50 years (69%). Roles within the cancer service comprised of nursing staff (44%), psychosocial staff (20%), medical staff (16%), administrative, technical support, and non-clinical managers (10%), and allied health and clinical trials staff (8%). Most staff were employed on a full-time basis (66%), with most employed in their current position for ≤ 2 years (42%).

**Table 2:** Staff demographic and professional characteristics

	**T0 (*n* = 106)**	
**Staff characteristics**	***n***	**%**
Age (in years)		
18–25	3	3
26–50	73	69
51–75	30	28
Gender		
Female	91	86
Male	15	14
Role^a^		
Nursing staff	47	44
Medical staff	17	16
Allied health & clinical trials staff	9	8
Administrative, technical support and non-clinical managers	11	10
Psychosocial staff	21	20
Missing	1	1
Employment Status		
Full-Time	70	66
Part-Time	36	34
Years of employment in current role		
≤ 2	45	42
2.01–5.00	19	18
5.01–10.00	17	16
≥ 10.01	25	24
Language spoken at home		
English	85	80
Non-English	21	20
Country of birth		
Australia	72	68
UK (England/Scotland/Wales/NI)	15	14
Other	19	18
Aboriginal or Torres Strait Islander		
No	106	100
Yes	0	0

^a^Roles included in the categories:

Nursing Staff: Nurse- RN/AIN, CNS, CNE Care Coordinator, CNC, NUM, Nurse Practitioner

Medical Staff: Oncologist, Haematologist, Psychiatrist, Registrar, Medical oncology Fellow

Allied Health & Clinical Trials Staff: Speech pathologist, Clinical Trials,

Administrative, technical support & non-clinical managers: Administrative, IT staff, Volunteer, Clinical Support Officer, Management, Program Coordinator, Practice Manager

Psychosocial staff: Psychologist, Psychologist Intern, Social Worker, Counsellor

### Survey scales

#### ORIC

An exploratory factor analysis was conducted using the T0 ORIC results. A single factor was identified (eigenvalue = 7.96). As we were unable to replicate the two-factor structure of the ORIC as in Shea et al. [16], the responses across all the ORIC items were summed for each respondent. The average total ORIC score was 47.52 out of a possible 60 (SD = 9.47, range = 16–60). The ratings for each ORIC item, averaged across services, are presented in Table 3. On average, items were rated positively.

**Table 3:** Mean ORIC ratings and the standard deviation for each item, averaged across services

ORIC item	Mean (SD)
1. Feel confident that the organisation can get people invested in implementing the anxiety and depression pathway	4.0 (0.99)
2. Are committed to implementing the anxiety and depression pathway	4.2 (0.89)
3. Feel confident that they can keep track of progress in implementing the anxiety and depression pathway	3.8 (0.97)
4. Will do whatever it takes to implement the anxiety and depression pathway	3.7 (1.08)
5. Feel confident that the organisation can support people as they adjust to implementing the anxiety and depression pathway	3.9 (0.98)
6. Want to implement the anxiety and depression pathway	4.3 (0.79)
7. Feel confident that they can keep the momentum going in implementing the anxiety and depression pathway	4.0 (0.91)
8. Feel confident that they can handle the challenges that might arise in implementing the anxiety and depression pathway	3.9 (0.98)
9. Are determined to implement the anxiety and depression pathway	3.9 (0.97)
10. Feel confident that they can coordinate tasks so that implementation goes smoothly	3.9 (0.94)
11. Are motivated to implement the anxiety and depression pathway	4.2 (0.98)
12. Feel confident that they can manage the politics of implementing the anxiety and depression pathway	3.7 (0.99)

#### Additional study-specific items

An exploratory factor analysis was conducted on the 13 study-specific additional items to assess their latent structure (see: [22]) (Additional file 1). Two factors were identified (eigenvalue > 1), accounting for 84.5% of the variance and were weakly correlated (0.29). Kaiser–Meyer–Olkin (KMO) Measure of Sampling Adequacy was acceptable at 0.77. Using a Varimax rotation (with Kaiser normalisation), most of the items showed acceptable loading onto the two factors identified, except for one item (“*Implementing the anxiety and depression pathway will cost the organisation too much money*”; factor loading = 0.54), and also an item which did not load onto any factor (“*The team evaluating the implementation of the anxiety and depression pathway have high credibility with me and I trust them*”), with both items excluded.

For the two factors identified, namely, *perceived benefit* and *perceived burden*, the scores within each factor were summed. The overall mean *perceived burden* scores were moderate (12.6, range: 5–20), while the mean *perceived benefit* scores were high (30.8, range: 15–35). The perceived benefit and burden scores were entered into the regression model to determine if perceived acceptability and appropriateness of the ADAPT CP predicted readiness scores. Refer to Additional file 1 for detail on what items loaded onto what factor, and for the mean ratings for each item, averaged across services. Internal consistency was acceptable (Cronbach’s Alpha for Factor 1 = 0.86 and for Factor 2 = 0.71).

### Identifying significant predictors of organisational readiness

#### Univariate analyses

Separate univariate regression analyses were conducted for each independent variable with the ORIC scores. With the significance criterion set to *p* < 0.25, the following variables were retained: number of treatment modalities, employment type, role, years in current role, perceived benefit, and perceived burden (Table 4).

**Table 4:** Univariate analyses assessing the association between each service- and staff-level factor on perceptions of readiness

	Average difference (B)^a^	95% CI	p^c^	Mean ORIC score
**Service-level factors**				
Location			0.36	
Inner regional	0	Referent		50.9
Major city	-3.5	-10.97 – 3.94		46.7
Size			0.63	
< 100	0	Referent		49.3
≥ 100	-1.7	-8.73 – 5.29		46.9
History of psychosocial screening			0.58	
No	0	Referent		46.8
Yes	1.8	-4.71 – 8.36		48.6
Number of treatment modalities			**0.02**	
1	0	Referent		45.4
2	-2.1	-8.82 – 4.72		44.0
3	4.8	-0.97 – 10.49		50.2
4	11.7	2.30 – 21.02		56.9
Number of streams			0.56	
1	0	Referent		45.4
2	-0.6	-13.50 – 12.24		44.3
≥ 3	3.6	-3.94 – 11.19		48.9
Service type			0.75	
Public	0	Referent		48.0
Private	-3.5	-16.20 – 9.18		44.3
Mixed	-3.5	-15.57 – 8.55		44.3
Clinical load of psychosocial staff	-0.00006	-0.0009 – 0.0008	0.89	–
**Staff-level factors**				
Age			0.26	
18–25 years	0	Referent		51.0
26–50 years	-2.8	-13.00 – 7.41		46.3
51–75 years	0.32	-10.13 – 10.77		50.2
Sex			0.58	
Female	0	Referent		47.7
Male	-1.4	-6.19 – 3.46		46.3
Employment type			**0.14**	
Full-time	0	Referent		48.7
Part-time	-2.7	-6.37 – 0.90		45.2
Role^b^			**0.06**	
Psychosocial staff	0	Referent		44.8
Medical staff	1.2	-4.25 – 6.59		45.9
Allied health & clinical trials staff	3.4	-3.39 – 10.20		49.8
Administrative, technical support and non-clinical managers	9.1	2.57 – 15.62		55.9
Nursing staff	1.0	-3.47 – 5.49		46.7
Years in current role			0.88	
≤ 2 years	0	Referent		48.2
2.01–5.00 years	0.7	-4.15 – 5.54		48.9
5.01–10.00 years	-1.4	-6.55 – 3.84		45.0
≥ 10.01 years	-1.1	-5.77 – 3.56		47.0
Perceived benefit			**0.001**	
	0.7	0.30 – 1.19		–
Perceived burden			**0.01**	
	0.9	0.22 – 1.48		–

^a^Mean difference: Positive values indicate on average higher ORIC scores, while negative values indicate on average lower ORIC scores. For categorical variables, these represent the average difference for a category when compared to the reference group. For continuous variables, these represent a 1-unit increase/decrease

^b^This univariate analysis was conducted with *N* = 105 due one cell missing the role information

^c^Bolded text indicates *p* < 0.25

#### Model building

Following a backward elimination regression, the final model containing only significant predictors of organisational readiness were the number of treatment modalities included (*p* = 0.01), role (*p* = 0.01), and perceived benefit (*p* = 0.001) (Table 5). Organisational readiness scores were significantly higher for services which implemented the ADAPT CP in four treatment modalities when compared to services that implemented ADAPT in one treatment modality (average difference = 9.4, 95% CI = 2.85 – 15.91, *p* = 0.01). Staff in administrative, technical support and non-clinical roles had significantly higher organisational readiness scores compared to psychosocial staff (average difference = 10.3, 95% CI = 4.23 – 16.40, *p* < 0.01). Higher organisational readiness scores were also significantly related to higher perceived benefit scores (average difference = 0.8, 95% CI = 0.34 – 1.28, *p* < 0.01).

**Table 5:** Regression results displaying the significant predictors of organisational readiness^a^

Factor	Average difference (B)	95% CI	*p*^b^
Number of treatment modalities			**0.01**
1	0	Referent	
2	-1.3	-5.45 – 2.89	
3	3.1	-0.80 – 7.04	
4	9.4	2.85 – 15.91	
Role			**0.01**
Psychosocial staff	0	Referent	
Medical staff	4.9	-0.65 – 10.51	
Allied health & clinical trials staff	5.0	-1.62 – 11.51	
Administrative, technical support and non-clinical managers	10.3	4.23 – 16.40	
Nursing staff	1.3	-3.13 – 5.67	
Perceived benefit			**0.001**
	0.8	0.34 – 1.28	

^a^This analysis was conducted with *N* = 105 due one cell missing the role information

^b^Statistical significance set to *p* < .05

Assumptions of multiple regression were assessed. Five cases were identified as potential outliers having studentised residuals that exceeded ± 2. However, these cases were retained to maximise power in an already small dataset, and because their inclusion and exclusion yielded the same pattern of results. The assumptions of normality of residuals and linearity between predictors and the outcome variable were violated for *perceived benefit*, with residuals skewed but this is likely due to the high perceived benefit (mean score of 30.8 out of a maximum of 35).

## Discussion

This study examined staff perceptions of their service’s level of readiness to implement the ADAPT CP, and identified factors associated with organisational readiness. As measured by the ORIC scale, readiness to implement change was relatively high, which may be attributed to the extensive engagement with services prior to implementation to recruit champions, tailoring of the clinical pathway (e.g., identify existing referral pathways, integrate the clinical pathway into workflows), staff training, and awareness raising activities. Alternatively, there may have been sample bias, with services readier to implement the ADAPT CP more likely to agree to participate in the study. While perceived readiness was relatively high, there remains an opportunity to further increase readiness and identify the generalisable learnings about what influences readiness for use in other contexts.

Holt et al. [8] proposed four types of factors that influence organisational readiness. Here, we examined staff- and service-level factors. Results revealed readiness for change was influenced by two staff-level factors; staff roles and individuals’ perceptions of the benefits of the ADAPT CP. Number of treatment modalities chosen by services in which to implement the ADAPT CP was identified as the only service-level factor associated with organisational readiness, with broader implementation associated with greater readiness.

We hypothesised that factors relating to psychosocial staff or the psychosocial care of patients would be associated with organisational readiness. Our hypothesis was only partly supported because, aside from staff role (which included staff in psychosocial roles) emerging as a significant predictor of organisational readiness, history of psychosocial screening, and clinical load of psychosocial staff were not significant predictors.

Staff in administrative, technical support and non-clinical roles had significantly higher organisational readiness scores compared to psychosocial staff. As the focus of the ADAPT CP was on the identification and management of distress and anxiety and depression in patients, this finding was surprising given the experience of psychosocial staff in providing psychological care. However, psychosocial staff may have felt more responsible for, and burdened by, the ADAPT CP than other staff, and thus less ready for this change. While the aim was for the ADAPT CP to be a multidisciplinary, whole-of-service initiative, with staff across various roles playing an important part in enacting the ADAPT CP within their service, across most services, more roles were allocated to psychosocial staff by lead team members [23] than to other roles or disciplines. Generally, psychosocial staff were responsible for introducing the ADAPT CP to patients, triaging patients after they completed distress screening, and monitoring referral uptake. This suggests that either greater resourcing of psychosocial teams within cancer services, or more even distribution of tasks across service roles or clinical disciplines would facilitate greater organisational readiness for a structured approach to psychosocial care. Other recent work suggests that an emotional affective commitment to change, that is, a desire to support the change, is a critical influence on organisational readiness [27]. These authors suggest that investing in improving interpersonal relationships with and between team members and improving communication to and from staff would facilitate greater co-operation and a reduced sense of burden, improving organisational readiness for change.

Positive attitudes toward the acceptability and appropriateness of the ADAPT CP were also associated with higher levels of perceived organisational readiness. Indeed, Weiner [4] proposes that commitment to change is largely dependent on the perceived value of the change. Specifically, the more that organisational members value the change, the more likely that they will want to implement the change. The reasons why individual members value the change need not be the same, so long as there is a general view that the change is valued [4]. Staff may value change that aligns with their service’s goals and mission and/or holds benefits for health service delivery and patient outcomes. Indeed, our participants indicated that the ADAPT CP was highly valued for these reasons, scoring on average 4.42/5 on the item: *The clinical pathway for anxiety and depression aligns with our organisation's mission and goals,* and 4.63/5 on the item: *Patients in our local service would benefit from treatment for anxiety and/or depression* (Additional file 1). These data reinforce the importance of providing staff with evidence of value and alignment with service goals prior to implementation, particularly as affective commitment to change is independently associated with individual and collective readiness [27].

Organisational readiness scores were significantly higher for services which implemented the ADAPT CP in four treatment modalities versus one. This suggests that staff who were confident in their service’s ability to implement the ADAPT CP were also confident to apply it in more than one treatment modality. As noted in a previous qualitative study which examined the rationale for what and why decisions were made to tailor the ADAPT CP across the 12 cancer services [23], some services opted for a more conservative approach initially to allow staff to familiarise themselves with the processes, with the plan to extend to additional treatment modalities or tumour groups once success had been achieved and confidence was higher. It would be of value to gather follow-up data to determine if this approach is successful, or rather complicates implementation which is piecemeal across the organisation. Alternatively, this may reflect a greater service-level commitment with more departments in agreement to implement change, or that those services placed a greater value in having more patients to benefit.

There are some potential limitations of the present study. The first is related to the survey response rates. In this study, few staff completed the surveys relative to the number of surveys sent. We cannot rule out the possibility that those who completed the survey may have been more engaged with the ADAPT program and held more positive views of the ADAPT CP, potentially introducing some level of bias. Thus, the high perceived benefit scores reported may not be representative of all staffs’ views. Additionally, as part of the larger program of work, the ORIC scale was also administered at the midpoint and endpoint of the implementation period (Additional file 2). However, staff attrition (changing positions or leaving the service) and drop-out meant that the sample sizes were too small to be able to conduct any meaningful longitudinal analyses. Nonetheless, this study provided valuable insights into the initial level of perceived readiness for change. Analyses over time may provide further insight into how initial readiness influences ongoing perceived readiness.

Second, we were able to examine only 2 out of the 4 types of factors proposed by Holt and colleagues as being influential to organisational readiness (staff and service-level factors) [8]. In addition, Holt suggests that the content of the change, and the process by which the change was implemented influence levels of organisational readiness. However, to examine these types of factors would have required comparing the ADAPT CP implementation with implementation of other types of CPs or interventions, which was beyond the scope of the current study.

This study did, however, have several strengths, including the size and complexity of the trial, its focus on organisational readiness both from a quantitative perspective here, and a qualitative perspective in our earlier paper [12]. Together, they are some of the very few psycho-oncology studies to provide a comprehensive exploration of factors impacting organisational readiness for change.

In conclusion, this study identified a high level of readiness for implementing an anxiety/depression CP in routine cancer care, in 12 cancer services. Factors associated with organisational readiness for change suggested the importance of adequately resourcing and supporting staff to implement change, effectively communicating the value of the change, and taking a whole-of-service approach to change. However, a potential sample bias may exist, therefore caution is needed in interpretating the results and assuming generalisability of the results. Future studies of complex multi-component interventions trials that test implementation strategies are needed to identify additional factors that may be associated with readiness and engagement with change processes.

### Supplementary Information

**Additional file 1.** Summary of the 13 study-developed additional item responses and their factor structure following the factor analysis (see: 22)*.*

**Additional file 2.** Mean ORIC ratings and the standard deviation for each item at T1 (6-months post-implementation) and T2 (12-months post-implementation), averaged across services.

## Data Availability

The datasets used and/or analysed during the current study are available from the corresponding author on reasonable request.

## Abbreviations

ADAPT CP: Clinical Pathway for the Screening, Assessment and Management of Anxiety and Depression in Adult Cancer Patients-Australian Guidelines
ADAPT: The Anxiety and Depression Pathway Program-A Translational Program Grant – A Sustainable and supported clinical pathway for managing anxiety and depression in cancer patients
ADAPT Portal: Web-based system to operationalise the Clinical Pathway for the Screening, Assessment and Management of Anxiety and Depression in Adult Cancer Patients: Australian Guidelines
AIN: Assistant in nursing
CI: Confidence interval
CNC: Clinical nurse consultant
CNE: Cancer nurse educator
CNS: Clinical nurse specialist
CP: Clinical Pathway
FTE: Full-time equivalent
KMO: Kaiser–Meyer–Olkin
NSW: New South Wales
NUM: Nurse unit manager
ORIC: Organizational Readiness for Implementing Change
RCT: Randomised Controlled Trial
REDCap: Research Electronic Data Capture
RN: Registered nurse
SD: Standard deviation
