# Supplementary material for: Staff- and service-level factors associated with organisational readiness to implement a clinical pathway for the identification, assessment, and management of anxiety and depression in adults with cancer
Source: BMC Health Serv Res. 2023 Aug 15;23:866. doi: 10.1186/s12913-023-09829-2 (PMC10426102; doi:10.1186/s12913-023-09829-2)
Supplement: Supplementary file 1 — Additional file 1. Summary of the 13 study-developed additional item responses and their factor structure following the factor analysis (see: 22). [file 12913_2023_9829_MOESM1_ESM.docx]

**Additional file**

***File name:*** Additional file 1

***File format:*** .docx

**Title of data:** Additional file 1: Summary of the 13 study-developed additional item responses and their factor structure following the factor analysis (see: 22)

**Description of data:** A table displaying the means and standard deviations of the 13 study-developed additional items and how each item loaded on to the two factors identified from the factor analysis.

Additional file 1: Summary of the 13 study-developed additional item responses and their factor structure following the factor analysis (see: 22)

|  | **Mean** | **SD** |
| --- | --- | --- |
| **Factor 1 – “Perceived benefit”** |  |  |
| Q1.Patients in our local service would benefit from treatment for anxiety and/or depression | 4.63 | 0.62 |
| Q2.There is high quality evidence that psychological interventions can reduce anxiety in cancer patients | 4.52 | 0.69 |
| Q3.There is high quality evidence that psychological interventions can reduce depression in cancer patients | 4.42 | 0.80 |
| Q4.The leaders in my organisation believe implementation of the anxiety and depression pathway is important | 4.12 | 0.85 |
| Q5.The leaders of this organisation who are driving implementation of the anxiety and depression pathway have high credibility with me and I trust them | 4.25 | 0.86 |
| Q9.I understand why the organisation needs to implement the clinical pathway for anxiety and depression | 4.62 | 0.52 |
| Q11.The clinical pathway for anxiety and depression aligns with our organisation's mission and goals | 4.42 | 0.62 |
| **Factor 2 – “Perceived burden”** |  |  |
| Q7.**Implementing the anxiety and depression pathway will increase my workload | 2.27 | 0.90 |
| Q8.**Implementing the anxiety and depression pathway will take up too much of my time | 3.10 | 0.85 |
| Q12.I am confident we have the necessary staff to implement the anxiety and depression pathway | 3.42 | 1.08 |
| Q13.I am confident we have enough resources to implement the anxiety and depression pathway | 3.31 | 1.01 |
| **Excluded items** |  |  |
| ^a^Q6.The team evaluating the implementation of the anxiety and depression pathway have high credibility with me and I trust them | 4.26 | 0.83 |
| ^b^Q10.**Implementing the pathway for anxiety and depression will cost the organisation too much money | 3.49 | 0.84 |

^a^ Item 6 loaded equally on both factors and has been omitted.

^b^ Item 10 did not load on any factor and has been omitted.

** Items were reverse-coded.
