# Supplementary material for: Staff- and service-level factors associated with organisational readiness to implement a clinical pathway for the identification, assessment, and management of anxiety and depression in adults with cancer
Source: BMC Health Serv Res. 2023 Aug 15;23:866. doi: 10.1186/s12913-023-09829-2 (PMC10426102; doi:10.1186/s12913-023-09829-2)
Supplement: Supplementary file 2 — Additional file 2. Mean ORIC ratings and the standard deviation for each item at T1 (6-months post-implementation) and T2 (12-months post-implementation), averaged across services. [file 12913_2023_9829_MOESM2_ESM.docx]

**Additional file**

***File name:*** Additional file 2

***File format:*** .docx

**Title of data:** Additional file 2: Mean ORIC ratings and the standard deviation for each item at T1 (6-months post-implementation) and T2 (12-months post-implementation), averaged across services

**Description of data:** A table displaying the means and standard deviations of the ORIC items 6- and 12-months post-implementation, averaged across the 12 services.

Additional file 2: Mean ORIC ratings and the standard deviation for each item at T1 (6-months post-implementation) and T2 (12-months post-implementation), averaged across services

| **ORIC item** | **Mean (SD)** | |
| --- | --- | --- |
|  | **T1**  **(n=58)** | **T2**  **(n=57)** |
| 1. Feel confident that the organisation can get people invested in implementing the anxiety and depression pathway | 3.4 (1.11) | 3.2 (1.24) |
| 2. Are committed to implementing the anxiety and depression pathway | 3.7 (0.99) | 3.4 (1.23) |
| 3. Feel confident that they can keep track of progress in implementing the anxiety and depression pathway | 3.6 (0.94) | 3.3 (1.10) |
| 4. Will do whatever it takes to implement the anxiety and depression pathway | 3.2 (1.17) | 2.8 (1.28) |
| 5. Feel confident that the organisation can support people as they adjust to implementing the anxiety and depression pathway | 3.5 (0.98) | 3.0 (1.32) |
| 6. Want to implement the anxiety and depression pathway | 3.9 (1.05) | 3.7 (1.14) |
| 7. Feel confident that they can keep the momentum going in implementing the anxiety and depression pathway | 3.2 (1.09) | 2.8 (1.19) |
| 8. Feel confident that they can handle the challenges that might arise in implementing the anxiety and depression pathway | 3.5 (1.00) | 3.0 (1.10) |
| 9. Are determined to implement the anxiety and depression pathway | 3.3 (1.09) | 3.1 (1.23) |
| 10. Feel confident that they can coordinate tasks so that implementation goes smoothly | 3.4 (1.04) | 3.0 (1.04) |
| 11. Are motivated to implement the anxiety and depression pathway | 3.6 (1.09) | 3.3 (1.22) |
| 12. Feel confident that they can manage the politics of implementing the anxiety and depression pathway | 3.2 (1.08) | 2.9 (1.13) |
| **TOTAL** | 41.6 (9.82) | 37.5 (11.03) |
